# 5-Bromo-2-iodo-1,3-dimethyl­benzene

**DOI:** 10.1107/S1600536807064537

**Published:** 2007-12-06

**Authors:** Rui Liu, Yu-Hao Li, Wei Luo, Shan Liu, Hong-Jun Zhu

**Affiliations:** a Department of Applied Chemistry, College of Science, Nanjing University of Technology, Nanjing 210009, People’s Republic of China

## Abstract

The asymmetric unit of the title compound, C_8_H_8_BrI, contains three independent mol­ecules. In each molecule, the Br, I and C atoms of the methyl groups lie in the benzene ring plane. Intra­molecular C—H⋯I hydrogen bonds result in the formation of three planar five-membered rings, which are nearly coplanar with the adjacent rings.

## Related literature

For general background, see: Hu *et al.* (2001 ▶). For bond-length data, see: Allen *et al.* (1987 ▶).
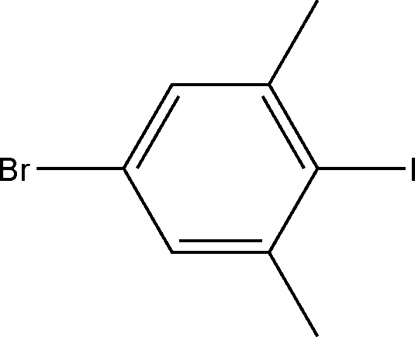


## Experimental

### 

#### Crystal data

C_8_H_8_BrI
*M* *_r_* = 310.94
Triclinic, 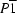

*a* = 10.282 (2) Å
*b* = 11.314 (2) Å
*c* = 12.951 (3) Å
α = 69.27 (3)°
β = 89.11 (3)°
γ = 83.70 (3)°
*V* = 1400.1 (6) Å^3^
*Z* = 6
Mo *K*α radiation
μ = 7.64 mm^−1^
*T* = 294 (2) K
0.10 × 0.10 × 0.10 mm

#### Data collection

Enraf–Nonius CAD-4 diffractometer
Absorption correction: ψ scan (North *et al.*, 1968 ▶) *T*_min_ = 0.466, *T*_max_ = 0.466
5802 measured reflections
5481 independent reflections
2809 reflections with *I* > 2σ(*I*)
*R*_int_ = 0.042
3 standard reflections frequency: 120 min intensity decay: none

#### Refinement

*R*[*F* ^2^ > 2σ(*F* ^2^)] = 0.059
*wR*(*F* ^2^) = 0.133
*S* = 1.06
5481 reflections
271 parameters
H-atom parameters constrained
Δρ_max_ = 0.76 e Å^−3^
Δρ_min_ = −0.72 e Å^−3^

### 

Data collection: *CAD-4 Software* (Enraf–Nonius, 1985 ▶); cell refinement: *CAD-4 Software*; data reduction: *XCAD4* (Harms & Wocadlo, 1995 ▶); program(s) used to solve structure: *SHELXS97* (Sheldrick, 1997 ▶); program(s) used to refine structure: *SHELXL97* (Sheldrick, 1997 ▶); molecular graphics: *PLATON* (Spek, 2003 ▶); software used to prepare material for publication: *SHELXTL* (Bruker, 2000 ▶).

## Supplementary Material

Crystal structure: contains datablocks I, global. DOI: 10.1107/S1600536807064537/hk2404sup1.cif

Structure factors: contains datablocks I. DOI: 10.1107/S1600536807064537/hk2404Isup2.hkl

Additional supplementary materials:  crystallographic information; 3D view; checkCIF report

## Figures and Tables

**Table 1:** Hydrogen-bond geometry (Å, °)

*D*—H⋯*A*	*D*—H	H⋯*A*	*D*⋯*A*	*D*—H⋯*A*
C1—H1*A*⋯I1	0.96	2.70	3.316 (11)	122
C10—H10*A*⋯I2	0.96	2.70	3.303 (10)	122
C18—H18*A*⋯I3	0.96	2.63	3.252 (10)	123

**Fig. 1.**
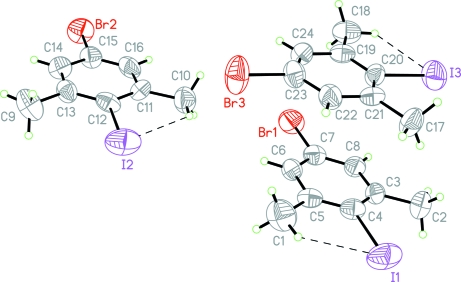
The molecular structure of the title molecule with the atom-numbering scheme. Displacement ellipsoids are drawn at the 50% probability level. Hydrogen bonds are shown as dashed lines.

**Fig. 2.**
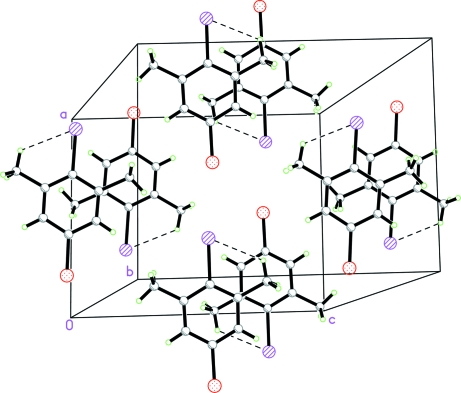
A packing diagram of (I). Hydrogen bonds are shown as dashed lines.

## supplementary crystallographic information

### Comment

The title compound, (I), contains two different halogen groups, which can react with different groups to prepare various function organic compounds by the different reaction activity of Br and I. It is a kind of aromatic organic intermediate that can be used for many fields such as aromatic conductive polymer, organometallic chemistry (Hu *et al.*, 2001). We herein report its crystal structure.

The asymmetric unit of (I) contains three independent molecules (Fig. 1), in which the bond lengths and angles (Table 1) are within normal ranges (Allen *et al.*, 1987). The Br, I and C atoms of the methyl groups lie in the benzene ring planes. The intramolecular C—H···I hydrogen bonds (Table 2) result in the formations of three planar five-membered rings; B (I1/H1A/C1/C4/C5), D (I2/H10A/C10—C12) and F (I3/H18A/C18—C20). Rings A (C3—C8), C (C11—C16) and E (C19—C24) are, of course, planar and the dihedral angles between them are A/B = 1.29 (3)°, C/D = 1.73 (3)° and E/F = 1.77 (2)°. So, the adjacent rings are also nearly co-planar.

As can be seen from the packing diagram, (Fig. 2), the molecules are stacked along the *b* axis. The π-π interactions of benzene rings with a face-to-face stacking distance of 3.636 Å are also found.

### Experimental

For the preparation of the title compound, a mixture of 4-bromo-2,6-dimethyl- aniline (4.0 g, 20 mmol), concentrated sulfuric acid (40 mmol, 2.24 ml) and water (100 ml) was stirred in an ice bath. When the mixture was below 278 K, the solution of sodium nitrite (1.75 g, 25 mmol) and water (100 ml) was added dropwise. Then, the mixture was added to a solution of KI (3.3 g, 20 mmol) and water (50 ml) with stirring. The solid residue was extracted with boiling hexane (40 ml) and hexane was distilled off. The product was recrystallized from ethanol. The crystals were obtained by dissolving (I) in ethanol (20 ml) and evaporating ethanol slowly at room temperature for about 10 d.

### Refinement

H atoms were positioned geometrically, with C—H = 0.93 and 0.96 Å for aromatic and methyl H, respectively, and constrained to ride on their parent atoms, with *U*_iso_(H) = *xU*_eq_(C), where *x* = 1.5 for methyl H, and *x* = 1.2 for aromatic H atoms.

### Crystal data

**Table:**

C_8_H_8_BrI	*Z* = 6
*M**_r_* = 310.94	*F*_000_ = 864
Triclinic, *P*1	*D*_x_ = 2.213 Mg m^−^^3^
Hall symbol: -P 1	Melting point: 307 K
*a* = 10.282 (2) Å	Mo *K*α radiation λ = 0.71073 Å
*b* = 11.314 (2) Å	Cell parameters from 25 reflections
*c* = 12.951 (3) Å	θ = 10–13º
α = 69.27 (3)º	µ = 7.64 mm^−^^1^
β = 89.11 (3)º	*T* = 294 (2) K
γ = 83.70 (3)º	Block, colorless
*V* = 1400.1 (6) Å^3^	0.10 × 0.10 × 0.10 mm

### Data collection

**Table:**

Enraf–Nonius CAD-4 diffractometer	*R*_int_ = 0.042
Radiation source: fine-focus sealed tube	θ_max_ = 26.0º
Monochromator: graphite	θ_min_ = 1.7º
*T* = 294(2) K	*h* = −12→12
ω/2θ scans	*k* = −12→13
Absorption correction: ψ scan(North *et al.*, 1968)	*l* = 0→15
*T*_min_ = 0.466, *T*_max_ = 0.466	3 standard reflections
5802 measured reflections	every 120 min
5481 independent reflections	intensity decay: none
2809 reflections with *I* > 2σ(*I*)	

### Refinement

**Table:**

Refinement on *F*^2^	Secondary atom site location: difference Fourier map
Least-squares matrix: full	Hydrogen site location: inferred from neighbouring sites
*R*[*F*^2^ > 2σ(*F*^2^)] = 0.059	H-atom parameters constrained
*wR*(*F*^2^) = 0.133	*w* = 1/[σ^2^(*F*_o_^2^) + (0.050*P*)^2^] where *P* = (*F*_o_^2^ + 2*F*_c_^2^)/3
*S* = 1.06	(Δ/σ)_max_ < 0.001
5481 reflections	Δρ_max_ = 0.76 e Å^−^^3^
271 parameters	Δρ_min_ = −0.72 e Å^−^^3^
Primary atom site location: structure-invariant direct methods	Extinction correction: none

### Special details

**Table:**

Geometry. All e.s.d.'s (except the e.s.d. in the dihedral angle between two l.s. planes) are estimated using the full covariance matrix. The cell e.s.d.'s are taken into account individually in the estimation of e.s.d.'s in distances, angles and torsion angles; correlations between e.s.d.'s in cell parameters are only used when they are defined by crystal symmetry. An approximate (isotropic) treatment of cell e.s.d.'s is used for estimating e.s.d.'s involving l.s. planes.
Refinement. Refinement of *F*^2^ against ALL reflections. The weighted *R*-factor *wR* and goodness of fit *S* are based on *F*^2^, conventional *R*-factors *R* are based on *F*, with *F* set to zero for negative *F*^2^. The threshold expression of *F*^2^ > σ(*F*^2^) is used only for calculating *R*-factors(gt) *etc*. and is not relevant to the choice of reflections for refinement. *R*-factors based on *F*^2^ are statistically about twice as large as those based on *F*, and *R*- factors based on ALL data will be even larger.

### Fractional atomic coordinates and isotropic or equivalent isotropic displacement parameters (Å^2^)

**Table:**

	*x*	*y*	*z*	*U*_iso_*/*U*_eq_	
I1	0.26694 (7)	0.30503 (8)	0.13847 (7)	0.0898 (3)	
I2	0.77050 (7)	0.30518 (8)	0.67167 (8)	0.0901 (3)	
I3	0.74437 (8)	−0.00666 (8)	0.03475 (6)	0.0798 (3)	
Br1	0.90853 (10)	0.36368 (12)	0.15368 (10)	0.0785 (4)	
Br2	1.40828 (10)	0.38917 (12)	0.62860 (10)	0.0782 (4)	
Br3	0.75298 (12)	0.00304 (14)	0.54991 (9)	0.0891 (4)	
C1	0.4494 (11)	0.3088 (11)	0.3459 (8)	0.086 (4)	
H1A	0.3588	0.3038	0.3332	0.129*	
H1B	0.4573	0.3817	0.3661	0.129*	
H1C	0.4847	0.2332	0.4046	0.129*	
C2	0.4834 (10)	0.3314 (10)	−0.0588 (8)	0.075 (3)	
H2A	0.5506	0.3421	−0.1127	0.113*	
H2B	0.4144	0.3999	−0.0856	0.113*	
H2C	0.4491	0.2520	−0.0459	0.113*	
C3	0.5415 (9)	0.3319 (8)	0.0497 (7)	0.050 (2)	
C4	0.4687 (8)	0.3180 (8)	0.1459 (8)	0.051 (2)	
C5	0.5241 (9)	0.3211 (8)	0.2418 (8)	0.051 (2)	
C6	0.6560 (9)	0.3308 (8)	0.2456 (7)	0.048 (2)	
H6A	0.6955	0.3292	0.3103	0.058*	
C7	0.7295 (9)	0.3430 (8)	0.1529 (8)	0.051 (2)	
C8	0.6722 (9)	0.3438 (8)	0.0557 (7)	0.054 (2)	
H8A	0.7234	0.3525	−0.0059	0.064*	
C9	0.9707 (9)	0.3346 (9)	0.8546 (8)	0.073 (3)	
H9A	1.0325	0.3445	0.9050	0.109*	
H9B	0.9362	0.2545	0.8875	0.109*	
H9C	0.9006	0.4024	0.8382	0.109*	
C10	0.9673 (10)	0.3129 (10)	0.4659 (8)	0.070 (3)	
H10A	0.8774	0.2996	0.4818	0.105*	
H10B	1.0117	0.2411	0.4518	0.105*	
H10C	0.9714	0.3883	0.4020	0.105*	
C11	1.0326 (9)	0.3283 (8)	0.5637 (7)	0.048 (2)	
C12	0.9713 (9)	0.3258 (8)	0.6616 (8)	0.053 (2)	
C13	1.0378 (10)	0.3385 (8)	0.7501 (7)	0.053 (2)	
C14	1.1692 (9)	0.3532 (8)	0.7396 (7)	0.055 (3)	
H14A	1.2160	0.3591	0.7982	0.066*	
C15	1.2316 (9)	0.3594 (9)	0.6444 (8)	0.055 (2)	
C16	1.1665 (9)	0.3466 (8)	0.5572 (8)	0.058 (3)	
H16A	1.2113	0.3502	0.4934	0.070*	
C17	0.5044 (9)	−0.0355 (10)	0.2075 (8)	0.068 (3)	
H17A	0.4380	−0.0454	0.2619	0.102*	
H17B	0.5147	−0.1090	0.1861	0.102*	
H17C	0.4790	0.0389	0.1440	0.102*	
C18	0.9888 (9)	0.0237 (10)	0.1763 (8)	0.072 (3)	
H18A	0.9751	0.0152	0.1062	0.108*	
H18B	1.0562	−0.0406	0.2178	0.108*	
H18C	1.0150	0.1062	0.1647	0.108*	
C19	0.8630 (9)	0.0082 (7)	0.2397 (7)	0.047 (2)	
C20	0.7480 (9)	−0.0034 (8)	0.1963 (7)	0.048 (2)	
C21	0.6322 (8)	−0.0216 (8)	0.2552 (7)	0.049 (2)	
C22	0.6335 (9)	−0.0184 (9)	0.3618 (8)	0.058 (3)	
H22A	0.5577	−0.0273	0.4028	0.070*	
C23	0.7527 (10)	−0.0012 (9)	0.4076 (7)	0.056 (3)	
C24	0.8632 (8)	0.0125 (8)	0.3466 (7)	0.047 (2)	
H24A	0.9400	0.0249	0.3765	0.056*	

### Atomic displacement parameters (Å^2^)

**Table:**

	*U*^11^	*U*^22^	*U*^33^	*U*^12^	*U*^13^	*U*^23^
I1	0.0426 (4)	0.0927 (6)	0.1290 (8)	−0.0114 (4)	0.0064 (4)	−0.0322 (5)
I2	0.0441 (4)	0.0918 (6)	0.1307 (7)	−0.0115 (4)	0.0106 (4)	−0.0342 (5)
I3	0.0972 (6)	0.0946 (6)	0.0534 (4)	−0.0077 (5)	0.0096 (4)	−0.0345 (4)
Br1	0.0446 (6)	0.0942 (9)	0.1125 (10)	−0.0111 (6)	0.0070 (6)	−0.0554 (7)
Br2	0.0446 (6)	0.0999 (9)	0.0907 (9)	−0.0137 (6)	0.0124 (6)	−0.0333 (7)
Br3	0.0792 (8)	0.1468 (12)	0.0609 (7)	−0.0395 (8)	0.0175 (6)	−0.0538 (8)
C1	0.085 (9)	0.101 (9)	0.076 (8)	−0.019 (7)	0.026 (7)	−0.034 (7)
C2	0.077 (8)	0.089 (8)	0.066 (7)	−0.011 (6)	−0.015 (6)	−0.034 (6)
C3	0.051 (6)	0.045 (6)	0.058 (6)	−0.006 (4)	−0.005 (5)	−0.023 (5)
C4	0.041 (6)	0.048 (6)	0.061 (6)	−0.005 (4)	0.009 (5)	−0.014 (5)
C5	0.043 (5)	0.048 (6)	0.057 (6)	0.002 (4)	0.008 (5)	−0.014 (5)
C6	0.049 (6)	0.055 (6)	0.041 (5)	−0.004 (5)	0.007 (4)	−0.019 (5)
C7	0.045 (6)	0.038 (5)	0.069 (7)	−0.010 (4)	0.003 (5)	−0.018 (5)
C8	0.042 (6)	0.069 (7)	0.053 (6)	0.000 (5)	0.005 (5)	−0.028 (5)
C9	0.070 (7)	0.084 (8)	0.078 (7)	−0.039 (6)	0.047 (6)	−0.040 (6)
C10	0.070 (7)	0.081 (8)	0.072 (7)	−0.014 (6)	−0.002 (6)	−0.039 (6)
C11	0.042 (5)	0.053 (6)	0.043 (5)	−0.005 (4)	−0.007 (4)	−0.010 (4)
C12	0.043 (6)	0.047 (6)	0.071 (7)	−0.006 (4)	0.016 (5)	−0.026 (5)
C13	0.067 (7)	0.048 (6)	0.043 (6)	−0.008 (5)	0.010 (5)	−0.017 (5)
C14	0.045 (6)	0.062 (6)	0.051 (6)	−0.011 (5)	0.014 (5)	−0.012 (5)
C15	0.036 (5)	0.071 (7)	0.062 (6)	−0.010 (5)	0.013 (5)	−0.029 (5)
C16	0.064 (7)	0.059 (6)	0.054 (6)	−0.002 (5)	0.020 (5)	−0.024 (5)
C17	0.049 (6)	0.093 (8)	0.068 (7)	−0.014 (6)	−0.009 (5)	−0.033 (6)
C18	0.047 (6)	0.082 (8)	0.084 (8)	0.001 (5)	0.018 (6)	−0.030 (6)
C19	0.045 (6)	0.034 (5)	0.050 (6)	−0.002 (4)	0.009 (4)	−0.002 (4)
C20	0.050 (6)	0.056 (6)	0.037 (5)	−0.003 (5)	−0.003 (4)	−0.017 (4)
C21	0.038 (5)	0.048 (6)	0.058 (6)	0.005 (4)	0.000 (5)	−0.015 (5)
C22	0.056 (6)	0.068 (7)	0.058 (6)	−0.004 (5)	0.005 (5)	−0.032 (5)
C23	0.067 (7)	0.061 (6)	0.043 (6)	−0.017 (5)	0.012 (5)	−0.021 (5)
C24	0.039 (5)	0.061 (6)	0.038 (5)	−0.007 (4)	0.009 (4)	−0.015 (4)

### Geometric parameters (Å, °)

**Table:**

I1—C4	2.102 (9)	C10—H10C	0.9600
Br1—C7	1.881 (9)	C11—C12	1.399 (12)
C1—C5	1.513 (13)	C11—C16	1.412 (12)
C1—H1A	0.9600	C12—C13	1.401 (12)
C1—H1B	0.9600	C13—C14	1.378 (12)
C1—H1C	0.9600	C14—C15	1.364 (12)
C2—C3	1.537 (11)	C14—H14A	0.9300
C2—H2A	0.9600	C15—C16	1.380 (12)
C2—H2B	0.9600	C16—H16A	0.9300
C2—H2C	0.9600	I3—C20	2.106 (8)
C3—C8	1.372 (11)	Br3—C23	1.861 (9)
C3—C4	1.413 (12)	C17—C21	1.508 (11)
C4—C5	1.387 (12)	C17—H17A	0.9600
C5—C6	1.376 (12)	C17—H17B	0.9600
C6—C7	1.382 (11)	C17—H17C	0.9600
C6—H6A	0.9300	C18—C19	1.515 (11)
C7—C8	1.395 (12)	C18—H18A	0.9600
C8—H8A	0.9300	C18—H18B	0.9600
I2—C12	2.100 (9)	C18—H18C	0.9600
Br2—C15	1.879 (9)	C19—C20	1.356 (11)
C9—C13	1.498 (12)	C19—C24	1.402 (11)
C9—H9A	0.9600	C20—C21	1.399 (11)
C9—H9B	0.9600	C21—C22	1.394 (12)
C9—H9C	0.9600	C22—C23	1.431 (12)
C10—C11	1.515 (12)	C22—H22A	0.9300
C10—H10A	0.9600	C23—C24	1.365 (11)
C10—H10B	0.9600	C24—H24A	0.9300
			
C5—C1—H1A	109.5	C11—C12—C13	122.8 (9)
C5—C1—H1B	109.5	C11—C12—I2	117.3 (7)
H1A—C1—H1B	109.5	C13—C12—I2	119.9 (7)
C5—C1—H1C	109.5	C14—C13—C12	118.1 (8)
H1A—C1—H1C	109.5	C14—C13—C9	119.9 (9)
H1B—C1—H1C	109.5	C12—C13—C9	122.0 (9)
C3—C2—H2A	109.5	C15—C14—C13	120.8 (9)
C3—C2—H2B	109.5	C15—C14—H14A	119.6
H2A—C2—H2B	109.5	C13—C14—H14A	119.6
C3—C2—H2C	109.5	C14—C15—C16	121.2 (9)
H2A—C2—H2C	109.5	C14—C15—Br2	120.2 (7)
H2B—C2—H2C	109.5	C16—C15—Br2	118.6 (7)
C8—C3—C4	117.2 (8)	C15—C16—C11	120.7 (8)
C8—C3—C2	118.9 (9)	C15—C16—H16A	119.6
C4—C3—C2	123.9 (9)	C11—C16—H16A	119.6
C5—C4—C3	122.3 (8)	C21—C17—H17A	109.5
C5—C4—I1	119.4 (7)	C21—C17—H17B	109.5
C3—C4—I1	118.2 (7)	H17A—C17—H17B	109.5
C6—C5—C4	119.0 (9)	C21—C17—H17C	109.5
C6—C5—C1	116.9 (9)	H17A—C17—H17C	109.5
C4—C5—C1	124.0 (9)	H17B—C17—H17C	109.5
C5—C6—C7	119.7 (9)	C19—C18—H18A	109.5
C5—C6—H6A	120.2	C19—C18—H18B	109.5
C7—C6—H6A	120.2	H18A—C18—H18B	109.5
C6—C7—C8	120.9 (8)	C19—C18—H18C	109.5
C6—C7—Br1	120.6 (7)	H18A—C18—H18C	109.5
C8—C7—Br1	118.4 (7)	H18B—C18—H18C	109.5
C3—C8—C7	120.9 (9)	C20—C19—C24	118.0 (8)
C3—C8—H8A	119.5	C20—C19—C18	123.2 (9)
C7—C8—H8A	119.5	C24—C19—C18	118.7 (8)
C13—C9—H9A	109.5	C19—C20—C21	123.6 (8)
C13—C9—H9B	109.5	C19—C20—I3	118.8 (7)
H9A—C9—H9B	109.5	C21—C20—I3	117.5 (6)
C13—C9—H9C	109.5	C22—C21—C20	117.8 (8)
H9A—C9—H9C	109.5	C22—C21—C17	118.3 (8)
H9B—C9—H9C	109.5	C20—C21—C17	123.8 (8)
C11—C10—H10A	109.5	C21—C22—C23	119.3 (8)
C11—C10—H10B	109.5	C21—C22—H22A	120.4
H10A—C10—H10B	109.5	C23—C22—H22A	120.4
C11—C10—H10C	109.5	C24—C23—C22	119.9 (8)
H10A—C10—H10C	109.5	C24—C23—Br3	121.3 (7)
H10B—C10—H10C	109.5	C22—C23—Br3	118.8 (7)
C12—C11—C16	116.3 (8)	C23—C24—C19	121.2 (8)
C12—C11—C10	125.5 (9)	C23—C24—H24A	119.4
C16—C11—C10	118.1 (8)	C19—C24—H24A	119.4
			
C8—C3—C4—C5	2.5 (13)	C12—C13—C14—C15	2.0 (14)
C2—C3—C4—C5	−178.9 (9)	C9—C13—C14—C15	−178.8 (8)
C8—C3—C4—I1	178.2 (7)	C13—C14—C15—C16	−2.3 (15)
C2—C3—C4—I1	−3.1 (12)	C13—C14—C15—Br2	176.4 (7)
C3—C4—C5—C6	−3.7 (14)	C14—C15—C16—C11	0.7 (15)
I1—C4—C5—C6	−179.4 (6)	Br2—C15—C16—C11	−177.9 (7)
C3—C4—C5—C1	179.0 (9)	C12—C11—C16—C15	0.9 (13)
I1—C4—C5—C1	3.3 (13)	C10—C11—C16—C15	−179.1 (9)
C4—C5—C6—C7	2.9 (14)	C24—C19—C20—C21	−5.4 (13)
C1—C5—C6—C7	−179.6 (8)	C18—C19—C20—C21	178.0 (9)
C5—C6—C7—C8	−0.9 (13)	C24—C19—C20—I3	178.9 (6)
C5—C6—C7—Br1	177.3 (7)	C18—C19—C20—I3	2.2 (12)
C4—C3—C8—C7	−0.4 (13)	C19—C20—C21—C22	4.8 (14)
C2—C3—C8—C7	−179.2 (8)	I3—C20—C21—C22	−179.4 (6)
C6—C7—C8—C3	−0.3 (14)	C19—C20—C21—C17	−179.5 (9)
Br1—C7—C8—C3	−178.6 (7)	I3—C20—C21—C17	−3.7 (12)
C16—C11—C12—C13	−1.1 (13)	C20—C21—C22—C23	−2.1 (14)
C10—C11—C12—C13	178.9 (9)	C17—C21—C22—C23	−178.1 (8)
C16—C11—C12—I2	177.9 (6)	C21—C22—C23—C24	0.3 (14)
C10—C11—C12—I2	−2.1 (12)	C21—C22—C23—Br3	179.9 (7)
C11—C12—C13—C14	−0.3 (14)	C22—C23—C24—C19	−1.0 (14)
I2—C12—C13—C14	−179.3 (7)	Br3—C23—C24—C19	179.5 (6)
C11—C12—C13—C9	−179.5 (8)	C20—C19—C24—C23	3.4 (13)
I2—C12—C13—C9	1.5 (12)	C18—C19—C24—C23	−179.8 (8)

### Hydrogen-bond geometry (Å, °)

**Table:**

*D*—H···*A*	*D*—H	H···*A*	*D*···*A*	*D*—H···*A*
C1—H1A···I1	0.96	2.70	3.316 (11)	122
C10—H10A···I2	0.96	2.70	3.303 (10)	122
C18—H18A···I3	0.96	2.63	3.252 (10)	123
